# Rapid measurement of ageing by automated monitoring of movement of *C. elegans* populations

**DOI:** 10.1007/s11357-023-00998-w

**Published:** 2023-11-08

**Authors:** Giulia Zavagno, Adelaide Raimundo, Andy Kirby, Christopher Saunter, David Weinkove

**Affiliations:** 1 Department of Biosciences, Durham University, Stockton Road, Durham, DH1 3LE UK; 2 Magnitude Biosciences Limited, NETPark Plexus, Thomas Wright Way, Sedgefield, Durham, TS21 3FD UK

**Keywords:** Healthspan, *C. elegans*, Ageing, Metrics, Lifespan, Automation

## Abstract

Finding new interventions that slow ageing and maintain human health is a huge challenge of our time. The nematode *Caenorhabditis elegans* offers a rapid in vivo method to determine whether a compound extends its 2 to 3-week lifespan. Measuring lifespan is the standard method to monitor ageing, but a compound that extends lifespan will not necessarily maintain health. Here, we describe the automated monitoring of *C. elegans* movement from early to mid-adulthood as a faster healthspan-based method to measure ageing. Using the WormGazer™ technology, multiple Petri dishes each containing several *C. elegans* worms are imaged simultaneously and non-invasively by an array of cameras that can be scaled easily. This approach demonstrates that most functional decline in *C. elegans* occurs during the first week of adulthood. We find 7 days of imaging is sufficient to measure the dose-dependent efficacy of sulfamethoxazole to slow ageing, compared to 40 days required for a parallel lifespan experiment. Understanding any negative consequences of interventions that slow ageing is important. We show that the long-lived mutant *age-1(hx546)* stays active for longer than the wild type but it moves slower in early adulthood. Thus, continuous analysis of movement can rapidly identify interventions that slow ageing while simultaneously revealing any negative effects on health.

**Supplementary Information:**

The online version contains supplementary material available at 10.1007/s11357-023-00998-w.

## Introduction

Ageing drug discovery faces specific and unique challenges [1]. One challenge is that there are no fast cell-based systems to test whether a compound slows ageing. Instead, cell culture–based approaches depend on measuring aspects of cell biology thought to be important in ageing, known as the ‘Hallmarks of Ageing’ [2, 3]. This approach limits the targets to the bounds of current knowledge and to interventions that work on properties detectable in cultured cells. Ageing involves interactions of multiple biological systems at several levels (organs, tissues, cells, molecules), which can only be seen in whole organisms. Experiments in mice are time consuming, expensive and constrained by ethical regulation. Thus, testing in a model organism such as the nematode *Caenorhabditis elegans*, which ages in weeks, provides the opportunity to gain data from compounds rapidly in an in vivo ageing system [4]. Another challenge in ageing drug discovery is that interventions would be given in the long term to healthy people and so must be completely safe. Any potential side effects should be detected as early as possible in the drug discovery process, and *C. elegans* provides a rapid early in vivo system to do so.

At least 83% of *C. elegans* proteins have human homologs [5], and its short lifespan allows an easier study of ageing and how a drug behaves in an aged organism. Genetic analysis in *C. elegans* was used to discover that the insulin/IGF-1 signalling (IIS) pathway modulates ageing across several species [6–8], showing that at least one mechanism of ageing is well conserved through evolutionary distance. *C. elegans* has also been used to show that many long-lived mutants experience physiological trade-offs that include reduced fecundity or delayed development [9–13]. These findings highlight that measuring lifespan alone is not sufficient to understand whether an intervention will be beneficial in humans. We also need to be aware of any negative consequences on health.

The manual *C. elegans* lifespan assay is accessible and easily transferable between research groups but can be difficult to standardise between laboratories [14]. The assay collects binary data on whether the worm is alive or not by checking for signs of movement. If a worm is not moving, then it is prodded with a platinum wire to look for a response. If there is no movement response, the animal is scored as dead. Technologies that enable automated image analysis can increase the amount of data collected in the same experiment while making more standardised measurements and saving labour. These technologies assess time of death by measuring movement on solid media [15, 16] or in microfluidic environments [17, 18]. See ‘Discussion’ and Table 1.

Lifespan is only one indirect measure of ageing. Functional parameters, such as fecundity, body bends and pharyngeal pumping, inform how health changes with age, providing measures of healthspan, and there are technologies that can measure these changes using imaging [19–21]. However, these technologies are either invasive, because animals need to be moved to a device for testing, or require animals to be kept in microfluidic devices throughout their adult lives and thus be in a liquid environment, which is known to produce different physiology to animals kept in a solid environment [22]. To better mimic movement on solid media, microfluidic technologies have incorporated micropillars to allow animals to weave through them [18, 23].

Here, we describe how health can be monitored non-invasively, on agar-filled Petri dishes with live bacterial lawns, which are the standard conditions for laboratory culture of *C. elegans*. This technology, which we have named the WormGazer™, monitors many Petri dishes simultaneously without moving the dishes or the cameras, using a design with scalability in mind. By monitoring the worms from the L4 stage (1 day before adulthood) and with an experimental runtime of 7–14 days into adulthood at 24°C, we show that measuring movement in this timeframe can detect improvements in health created by drugs that slow ageing. We further use the drug sulfamethoxazole (SMX) to show that delays to movement decline are clear in the first 7 days of imaging, whereas effects on survival in a concurrent parallel manual experiment take several times longer. Finally, we test the reported lifespan-extending compound alpha-ketoglutarate. This approach can be used to accelerate drug discovery by providing fast information about the effect of a compound in a whole organism, allowing more informed decisions about taking lead candidates forward.

## Results

### Monitoring the movement of large populations of worms over time

Multiple technologies have been designed to monitor worm movement. They mostly involve samples of worms (Petri dishes, multi-well plates or microfluidic devices) that need to be moved under a single fixed high-power camera in sequence to record images or videos, or an array of stationary worm samples where a camera moves between them (see ‘Discussion’ and Table 1). These technologies present the challenges of precisely and consistently aligning moving parts. To monitor large numbers of worms simultaneously, we use an array of low-grade cameras each connected to its own single-board computer. This distributed computing approach with no moving parts is designed to be robust and scalable. For example, hundreds of single-board computers can be connected to a single gateway computer using Ethernet, whereas connecting lots of cameras to PCs using USB has lower limits of scaling and creates more issues with space, cabling, cooling and data handling.

**Fig. 1:**
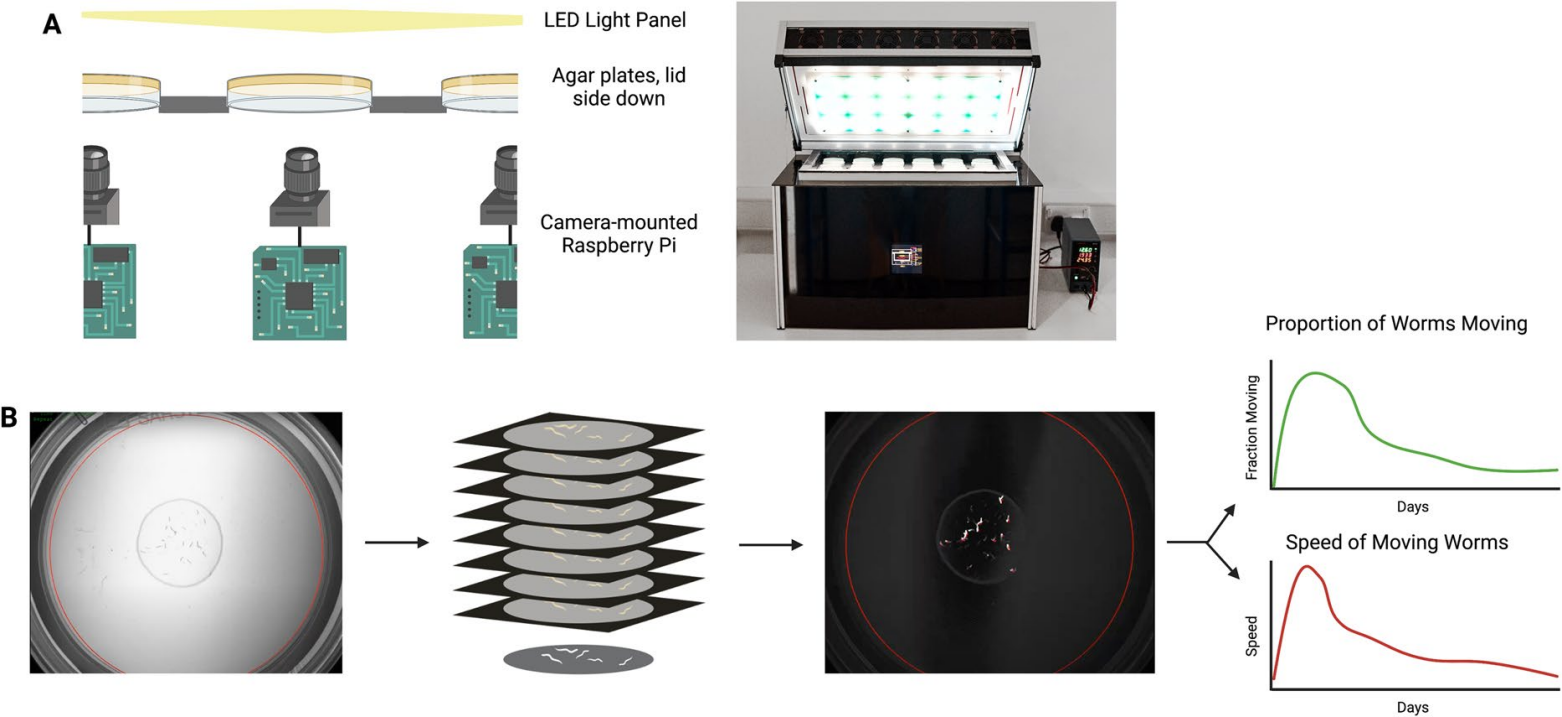
Schematic of the WormGazer™. **A** Petri dishes are placed in a temperature-controlled box with one imaging station per Petri dish. This diagram represents how the camera is positioned to take images and is connected to a single-board computer. The camera takes an image every 0.8 s for 160 s and repeats this process every 5 min. The first level of image processing occurs on the single-board computer. **B** A transmission image from the camera of a plate with 30 L4 worms (left). The images’ pixel values are averaged across 200 images in each imaging window (middle left), producing a high-contrast image where movement can be detected (middle right). The fraction of animals moving, and speed which the moving objects move are two main outputs (right)

The WormGazer™ is designed to use the same agar Petri dishes as used in standard *C. elegans* laboratory culture. Each 6-cm Petri dish is illuminated from above by permanently-on white LED panels and imaged by a camera below it (Fig. 1A). This camera is controlled by its connected single-board computer. As temperature is an important variable for ageing in *C. elegans* and since changes in temperature can affect the moisture content of the Petri dishes, the chamber containing the dishes is carefully temperature controlled to within ± 0.1°C using a feedback system of cooling by blowing externally chilled air (from an air-conditioned room for example) to counteract the heat emitted by the lights and electronics. Extra heating is provided by small resistance heaters when necessary.

Every 5 min, each camera records 200 images in a 160 s time window (an image every 0.8 s). The contrast between the worm and its translucent medium allows for the measurement of changes in pixel values (Fig. 1B). When the images from an imaging window are averaged and subtracted from each frame, and then overlaid, a high-contrast image is created, representing the movement in the imaging window, where pixels which changed in value during the imaging window due to movement are denoted as white and the unmoving background denoted in black (Fig. 1B). Where and how far a worm moves during the imaging window is therefore determined by following the path of these bright pixels at their centre of mass [24]. A threshold speed for detection is set to 10 µm s^−1^ to limit noise from other changes to the plate which the camera can pick up, such as worm trails in the bacterial lawn. There are some false positives occasionally resulting in apparently more than 100% worms moving at a single time point, but these anomalies are not at a sufficient level to mask movement at a population level.

All objects moving above the threshold are used to measure the fraction of worms moving (number of objects) and the mean speed of moving worms (length of objects) (Fig. 1B). The position of the worm on the plate (coordinates of object) is also recorded. There is an area limit for worm detection, denoted by the red ring (Fig. 1B). Using a series of sequential images to detect movement, this system can identify worms that move during the imaging window. It is not necessary to track each individual worm because measuring changes in behaviour at a population level is sufficient to measure ageing. Data analysis occurs on locally connected servers, and Python scripts are used to create initial data outputs.

Once an experimental run is started, the boxes are left undisturbed for the duration of the run, which is between 7 and 14 days. The worms are placed on the dishes at the L4 stage, and the box normally runs at 24.0°C. Under these conditions, wild-type worms become adults by 24 h, show a peak in movement around 48 h and then begin their ageing-associated decline in movement and speed. This system allows for the non-invasive monitoring of worms in their normal laboratory setting.

### Characterisation of the movement of the long-lived *age-1(hx546)* mutant

To test whether the system could detect changes due to ageing in *C. elegans* mutants established to show effects on lifespan, we compared the movement of the long-lived *age-1(hx546)* and the short-lived *daf-16(mu86)* mutants with a wild-type (WT) control. A minimum of 298 worms across ten Petri dishes per condition were compared over a 12-day period. Two micromolars of floxuridine (FUdR) was added to the media to prevent eggs from hatching.

**Fig. 2:**
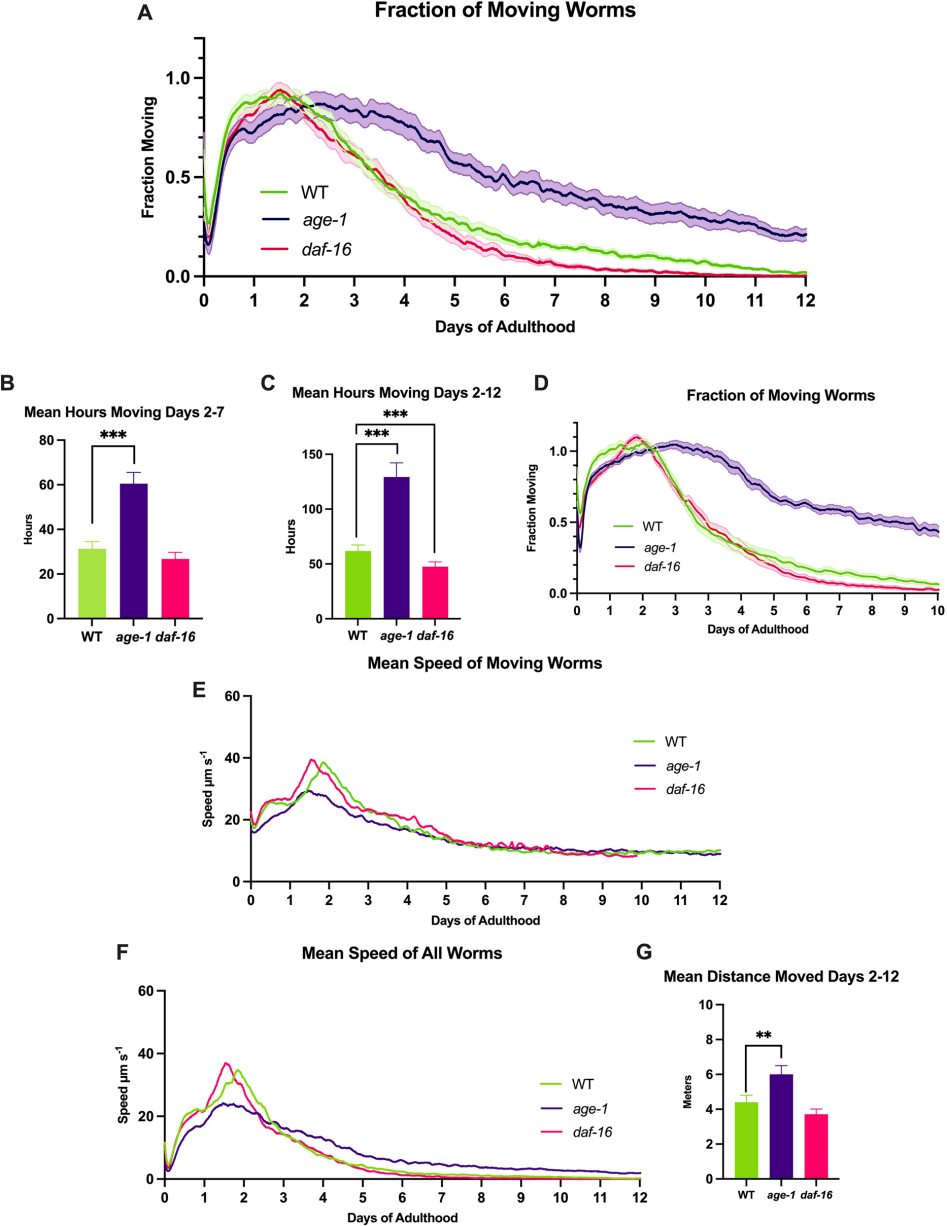
Movement analysis of *age-1*, *daf-16* and WT worms over 12 days. **A** The fraction moving graph shows the proportion of worms moving during the imaging window with SEM shading. *n* ≥ 298 worms, 10–12 Petri dishes per condition. **B** The area under the curve integration for days 2–7. **C** The area under the curve integration for days 2–12. **D** Fraction moving of an independent repeat, with SEM shading. *n* ≥ 420 worms, 14 Petri dishes per condition. **E** The mean speed of moving worms for **A**. **F** The mean speed of all worms, which is a function of **A** and **E**. **G** The area under the curve integration for days 2-12 for the mean speed of all worms (**F**). Petri dishes with 2 µM FUdR on DM. ** = *p* < 0.01, *** = *p* < 0.002, one-tailed test

The fraction of worms moving at any time is defined as the proportion of the population that moves during the 160 s imaging window. The lines of the graph are smoothed, with shading of the standard error of the mean (SEM) (Fig. 2A). Movement of the worms reaches a peak around day 2 so this time point is considered to be the start of age-related decline and is used as the starting point for the area under the curve (AUC) calculation, which produces the average time the worms spent moving in that time period.

The *age-1* mutant worms spend 93.5% more time moving between days 2 and 7 compared to the WT (Fig. 2B), and the worms continue to show an increased fraction moving until the end of the experiment (Fig. 2A). A video showing the movement in two representative Petri dishes illustrates this result and how the technology works (Supplementary Video S1). Interestingly at an early stage of adulthood (days 0.5 to 2), when ageing is likely to be insignificant, *age-1* mutants show a 12.1% lower fraction moving compared to WT (*p* < 0.05, Supplementary Table S1). This unexpected result suggests that the *age-1* mutant has fewer moving worms in early adulthood compared to the WT.

The *daf-16* mutant showed no significant difference in average time spent moving in the first 7 days when compared to the WT, but when the AUC is calculated for days 2 to 12, it spent 23.2% less time spent moving compared to WT (Fig. 2C). Overall, these results are consistent with slower ageing in the *age-1* mutant because it stays active for longer than the WT, while *daf-16* experiences a faster decline than the WT. The data show excellent reproducibility (Fig. 2D).

The mean speed of moving worms is a function of how far each detected worm moves during the imaging window (distance/time = speed, Fig. 2E) and does not consider those worms which do not move during the window. It is independent of the fraction of worms moving. In this analysis, the *age-1* mutant had a 27% lower speed than WT at day 2 (*p* < 0.002, Supplementary Table S2), as well as reaching its maximum mean speed at day 1.53 compared to day 1.94 for the WT (Fig. 2E). Meanwhile, there was no significant difference in speed between WT and *daf-16*. After day 10, no more speed data are available for *daf-16* as at least one entire dish of worms had zero worms that moved above the detection threshold of 10 µm s^−1^.

When the “fraction moving” and “mean speed of moving worms” parameters are multiplied, it produces the “mean speed of all worms” (Fig. 2F). This graph produces a representation of how quickly all animals moved in the experiment. In this case, it shows that *age-1* mutants have a lower peak in speed but continue moving at higher and faster levels for longer, and shows that *daf-16* mutants have an earlier and steeper decline in speed than the WT. The area under these curves represents the average distance the worms move and shows that *age-1* mutants moved a significantly greater distance than the WT but *daf-16* mutants were not significantly different from the WT (Fig. 2G). A full comparison of the two repeats can be found in Supplementary Fig. S1.

### Automated movement analysis vs manual lifespan

To compare movement analysis between the WormGazer™ and a manual lifespan assay, both approaches were performed in parallel. Twenty-six dishes of 30 temperature-sensitive sterile *glp-4(bn2)* worms per condition were prepared with 50% analysed on the WormGazer™ and 50% by manual lifespan methods (*n* = 390 worms set up per condition, per method). Worms were transferred to fresh dishes on days 7 and 14 with both methods. SMX was used as a positive control. SMX extends *C. elegans* lifespan in a dose-dependent manner up to at least 256 µg/mL by inhibiting folate synthesis in OP50 *Escherichia coli*. Inhibiting bacterial folate synthesis inhibits an *E. coli* activity that accelerates ageing [25]. In this experiment, we used low concentrations of SMX that, in a previous study, increased lifespan with an effect that was significant (4 µg/mL and 8 µg/mL) or not significant (1 µg/mL) [26].

**Fig. 3:**
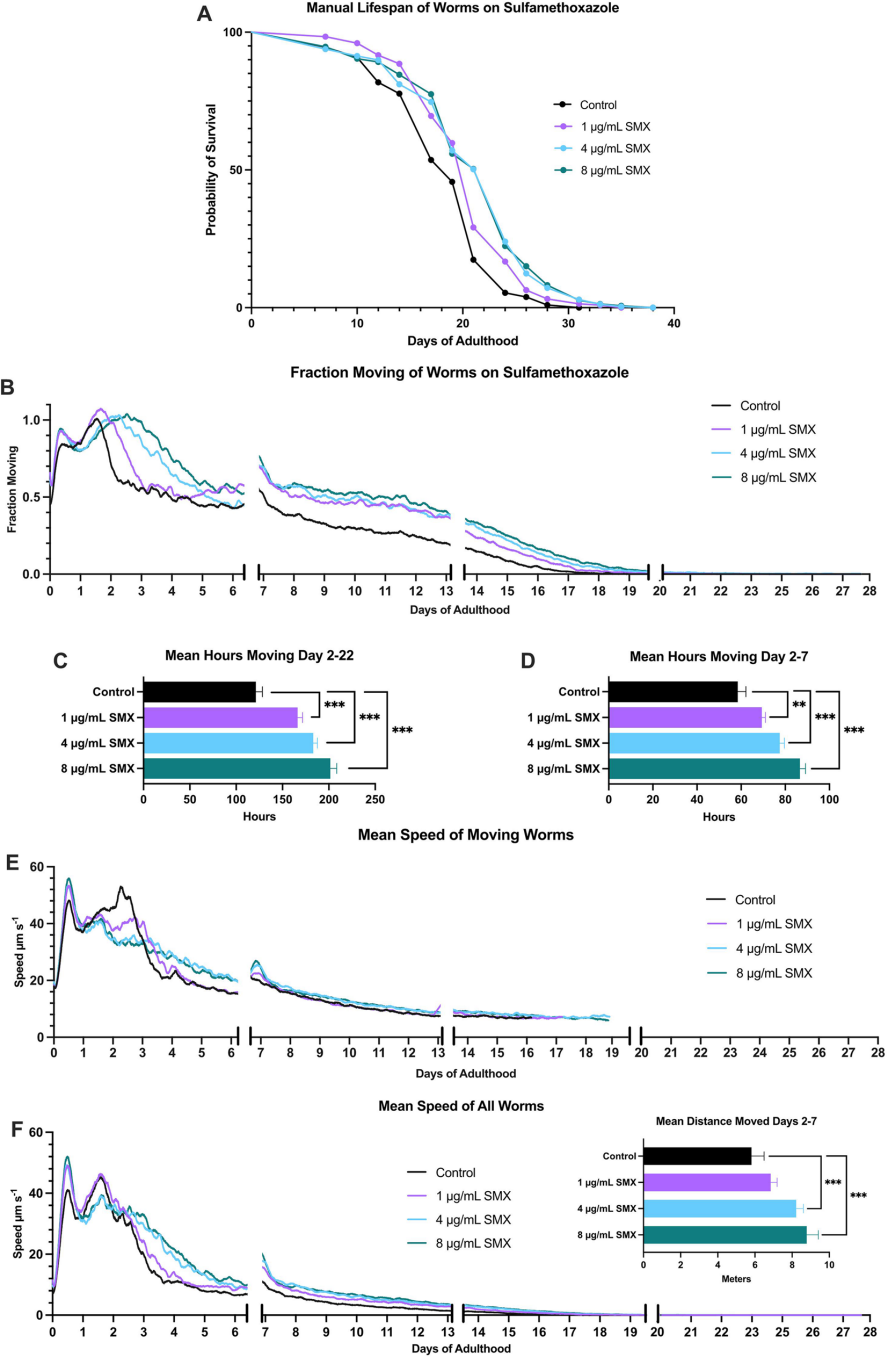
Manual lifespan versus WormGazer™ movement analysis. *glp-4* sterile worms were placed on as L4s and were transferred on days 7 and 14, being placed either into the WormGazer™ or into the 24°C incubator. **A** Survival curve of the manual lifespan method. **B** Fraction moving of the worms on the WormGazer™. An interrupted axis has been added to the fraction moving plot to indicate when the worms were transferred in the first two instances, while the last gap indicates when the machine was restarted. All these instances created bumps in movement which were smoothened by omitting 46 imaging periods. **C** Integration of the area under the curve is shown for days 2–22 and **D** days 2–7. **E** The mean speed of moving worms and **F** the mean speed of all worms which multiply **B** and **E**. Manual scoring occurred every other day on weekdays from day 7 onwards. ** = *p* < 0.01, *** = *p* < 0.002, one-tailed test. Compound added to DM agar. *n* ≥ 260 worms, 12 Petri dishes per condition, per technique

The manual lifespan experiment (Fig. 3A) showed a statistically significant increase in survival with increasing SMX concentration (*p* < 0.0001 for all conditions, Wilcoxon test). Mean survival for 1 µg/mL SMX was 20 days, while that for 4 µg/mL and 8 µg/mL was 21 days compared to control which was 18 days (Supplementary Table S1). This experiment took 40 days to complete.

In the WormGazer™ assay, worms showed an improvement in movement levels on all SMX concentrations (Fig. 3B) that was statistically significant from day 2 onwards (*p* < 0.002) (Fig. 3C). Notably, by day 7, the difference was already clear. The AUC between days 2 and 7 showed that all SMX concentrations led to significant improvements in healthspan (*p* < 0.01 for 1 µg/mL SMX, *p* < 0.002 for 4 µg/mL and 8 µg/mL SMX) (Fig. 3D). The automated monitoring also collected speed data (Fig. 3E, F), showing that the mean speed of moving worms was significantly slower for the SMX conditions at day 2.5 (*p* < 0.002, Supplementary Table S3) but speed across the whole experiment was significantly increased with SMX (Fig. 3F, inset).

The WormGazer™ experiment required disturbing the worms only three times over the time period while the manual lifespan required doing so every other day from day 7 onwards. No movement above the automated movement threshold was detected after day 22, whereas the manual method could detect smaller movements and response to prodding, and so lasted until day 40.

Overall, the two methods showed comparable results but with large differences in user effort and the amount of data captured. An AUC from days 2 to 7 showed the same significant result as a 40-day manual lifespan, which indicates that a 7-day healthspan can be used to detect lifespan extensions without requiring the work and time of a lifespan assay.

### Testing alpha-ketoglutarate

**Fig. 4:**
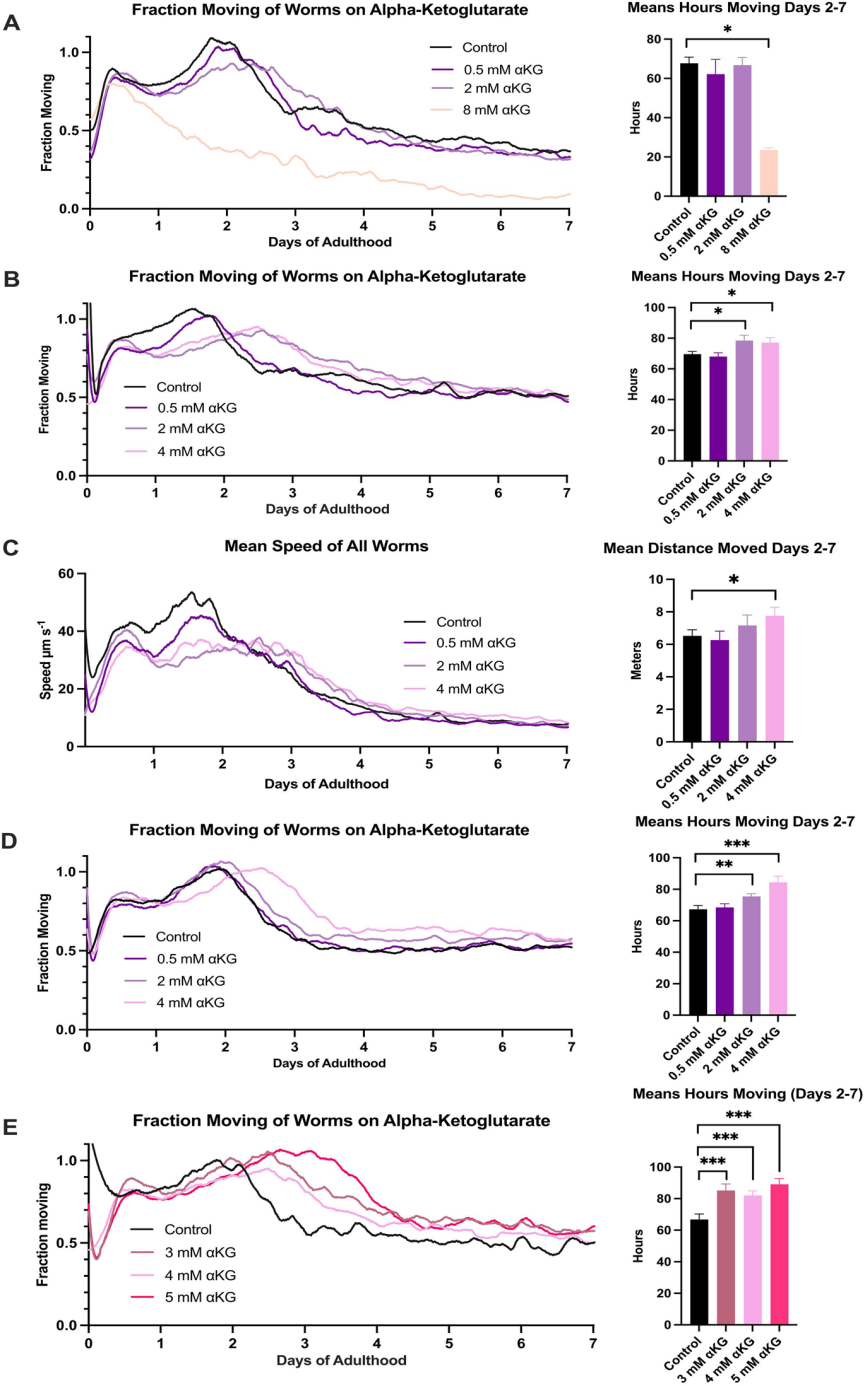
Alpha-ketoglutarate improves worm health. **A** Fraction moving of worms on alpha-ketoglutarate and its integration of the area under the curve (inset). *n* ≥ 180 worms, 6–8 Petri dishes per condition. **B** Fraction moving of a smaller concentration range, identifying two positive concentrations with integration of the AUC (inset). *n* ≥ 330 worms, 11–12 Petri dishes per condition. **C** Mean speed of all worms for the **B** experiment and integration of AUC (inset). **D** Fraction moving of a repeat for **B**, showing the same results with integration of AUC (inset). *n* ≥ 300 worms, 10–11 Petri dishes per condition. **E** Fraction moving of a smaller concentration range, identifying three positively effecting concentrations with integration of AUC (inset). *n* ≥ 150 worms, 5–9 Petri dishes per condition. * = *p* < 0.05, ** = *p* < 0.01, *** = *p* < 0.002, one-tailed test. Compound added to DM agar. Temperature-sensitive sterile *glp-4* worms were used

Next, we tested alpha-ketoglutarate (αKG), a metabolic intermediate in the Krebs cycle shown to extend lifespan in *C. elegans* [27]. To understand the effect of αKG on ageing, we conducted an experiment to look for a concentration-dependent effect on healthspan from 0.5 to 8 mM, with 8 mM being the reported effective concentration. We found that 8 mM, in fact, significantly reduced worm movement while 0.5 mM and 2 mM had no effect (Fig. 4A).

We tightened the concentration range to 0.5 to 4 mM and used higher animal numbers, to find that 2 mM and 4 mM αKG significantly improved movement by 12.7% and 10.7%, respectively, in the first 7 days compared to control (*p* < 0.002, Fig. 4B). Furthermore, 4 mM improved the mean speed of all worms (Fig. 4C). The mean speed of moving worms for all experiments can be found in Supplementary Fig. S2. This finding was reproducible (Fig. 4D). An even smaller concentration range was then assayed to find that 3 mM and 5 mM αKG were also effective in improving movement (Fig. 4E). Overall, by using a 7-day automated monitoring experiment, we found αKG is effective in maintaining health across a small range of concentrations.

## Discussion

### Population movement to measure ageing

In this paper, we have shown that *C. elegans* movement data can be used to measure ageing and produce results useful for assessing compounds and mutants within 7 days. This approach is made possible by the novel WormGazer™ automated imaging technology presented here, but other technologies that monitor movement across a large number of worms non-invasively and simultaneously could be used to achieve a similar result. We show that population-level metrics are sufficient to provide a fast method to measure ageing, and that it is not necessary to solve the considerable challenge of tracking individual worms. The traditional manual lifespan assay also does not track individual worms throughout their life, and it has been used successfully for decades because of the relative ease and transferability of the method.

A universal feature of ageing is heterogeneity. For example, within any *C. elegans* lifespan experiment, there is a large time difference between the death of the first worm and the last worm. Thus, the number of worms assayed is a very important factor to the success of the lifespan assay [28] and, similarly, the number of worms used is an important parameter for any measure of ageing. The WormGazer™ technology allows large numbers of worms to be set up, in a similar way that they are for a lifespan assay, but after the setup and apart from a post-run inspection, no further manual work is required and the data is available automatically.

The ubiquity of the lifespan assay has led to lifespan to be referred to almost synonymously with ageing. However, *C. elegans* lose optimal health within days of beginning their adult life, and thus ageing occurs long before death. The last week of a worm’s life is spent hardly moving at all and not eating [28]. Maintaining health and/or preventing chronic disease is the goal of much research into ageing as well as drug development in the field, and thus an assay that addresses these endpoints directly is important. The movement data gathered by the WormGazer™ does not need to be taken in isolation or have to replace lifespan analysis. Instead, it provides complementary endpoints. The non-invasive nature of the WormGazer™ technology means that, after the healthspan analysis, worms can be transferred to fresh Petri dishes and manual lifespan analysis (or other assays) can be performed on the same animals.

### Comparison with other technologies

Other technologies have been designed to measure ageing (Table 1). Briefly, the first major difference is that the WormGazer™ uses standard agar Petri dishes and standard worm culturing methods to set up, making it able to monitor worm behaviour in the same way as the majority of previous *C. elegans* research. Furthermore, it has no moving parts (as found in the WormBot, HeALTH or *C. elegans* observatory), which introduces the potential for variation between observations, or need to be moved under a camera/microscope (CeLab). It has a built-in temperature controller focussed on the worm samples rather than relying on external temperature control (as in all except HeALTH). The WormGazer™ images all worm samples in parallel (as opposed to sequential for CeLab, WorMotel, WormBot, HeALTH and Lifespan Machine). Finally, it requires no post-run data curation other than censoring Petri dishes with any quality control fails (e.g. contamination). Together, these features of the WormGazer™ make the technology very compatible with *C. elegans* ageing research and, therefore, very practical to use to monitor ageing under a wide variety of conditions.

**Table 1:** Technologies that automatedly measure ageing in *C. elegans*

Name	Liquid/agar?	Moving hardware?	Temperature control method	Parallel or sequential imaging?	Frequency of image capture	Manual post-run data curation required?
wMicroTracker [29]	Liquid	None	Via external incubator	Parallel	15 fps for 20 s every minute	Yes
NemaLife [18]	Liquid & micropillars Microfluidics	None	Via external incubator	Parallel	10 fps for unknown time	Yes
CeLab [30]	Liquid & micropillars Microfluidics	Dishes	Via external incubator	Sequential	Daily manual scoring with *Ce*Aid	Yes
WormBot [31]	Agar in 12-well plates	Camera	Room conditions	Sequential	30 fps for 1–5 min once a day	No
*C. elegans* observatory [32]	Agar in 60-mm dishes	Dish trays	Via adapted external incubator	Sequential	50 fps for 10 min every 6 h	No
HeALTH [33]	Liquid	Camera	Integrated in hardware	Sequential	14 fps for 10 s twice an hour	No
WorMotel [16]	Agar in PDMS mini-wells	Dish trays	Via external incubator	Sequential	0.2 fps continuously or for 30 min twice daily	No
WormGazer™	Agar in 60-mm dishes	No	Integrated into hardware	Parallel	1.25 fps for 160 s every 5 min	No
Lifespan Machine [15]	Agar in 50-mm plates	No	Via external incubator	Sequential	Twice in 2 h at 15-min and 105-min intervals	Yes

This table aims to showcase the range of automated hardware and software technologies which have been reported in the literature. Parallel imaging was judged based on whether all animals were being monitored at the same time point. Manual post-run data curation was defined as the requirement to carry out statistical or image analyses manually. Technologies where data only needed to be manually uploaded to a software pipeline, or the pipeline required manual activation to start, were not counted as requiring manual post-run curation.

### Validation and reproducibility

In this paper, we validated the approach of using movement to measure ageing by showing that the long-lived *age-1* mutant maintains movement with age longer than the wild type as measured by our assay. Simultaneously, we revealed features of early life movement of this mutant that are consistent with the idea that there are trade-offs in IIS mutants that increase lifespan [8].

Any method that measures ageing must be reproducible. Many environmental and epigenetic factors can affect health so it is critical to have very consistent culture conditions and worm preparation regardless of the method of measuring ageing. Because the WormGazer™ monitors movement from the beginning of the experiment, any anomalies in the worm behaviour can be detected before age-related decline begins, assisting quality control of the assay preparation. We have shown that the results from the WormGazer™ have good reproducibility (Figs. 2 and 4, Supplementary Fig. S1). The software and principles behind the WormGazer™ technology are available in a patent [24], and the technology can be accessed as a commercially available service at Magnitude Biosciences, making it accessible to those without experience with *C. elegans*.

### Utility in drug development to slow ageing

Using the WormGazer™ technology, it took only 7 days to see the age-slowing effect of SMX. We saw significant improvements with αKG after narrowing down from a wide concentration range. This finding illustrates the point that several concentrations of a compound should be tested to present the full picture, and that automated imaging over 7 days makes dose–response studies much faster and easier than using lifespan assays. The scalability of the technology coupled with an automated workflow to produce the Petri dishes and worms in a standardised way allows large numbers of new compounds to be screened at multiple concentrations or in combinations to find new interventions that slow ageing and improve human health. Once compounds that slow ageing have been identified, the power of *C. elegans* genetics can then be used with the same technology to uncover the mechanism of action. In the process of drug development to slow ageing, safety is a very important factor. Any drug that is given to healthy people over the long term must be very safe, and monitoring movement throughout early to mid-adulthood highlights any potential for negative effects. Overall, we show that monitoring the movement of large populations of worms with time is a powerful tool to address the challenges of discovering new drugs that slow ageing. These experiments therefore act as a proof-of-concept for using movement to measure health and represent one potential usage of such monitoring technology which could also be expanded to be used in behavioural, chemotaxis or toxicity assays.

## Materials and methods

### Worm maintenance

All strains were obtained from the Caenorhabditis Genetics Center (CGC) which is funded by the NIH Office of Research Infrastructure Programs (P40 OD010440). The strains used were N2, TJ1052 (*age-1(hx546)* II), CF1038 (*daf-16(mu86)* I) and SS104 (*glp-4(bn2)* II). *glp-4(bn2)* worms are temperature-sensitive sterile and were maintained at a permissive temperature of at 15°C. For consistency and convenient timing, all other strains were maintained at the same temperature.

To prepare for experiments on the WormGazer™, gravid worms are placed onto 9-cm Petri dishes to lay eggs 4 days before being placed on the machine. The mothers are removed after 48 h, and 24 h later, these 9-cm Petri dishes are shifted to 24°C to ensure large numbers of L4s or to induce sterility in the case of SS104 *glp-4(bn2)* worms. On the following day, worms at the L4 stage are selected and 30 picked onto each 6-cm Petri dish. At least six Petri dishes are prepared per condition. The edges of these dishes are covered with parafilm to prevent desiccation, but a 5-mm gap is left for air exchange. Plates are then loaded into the machines and left undisturbed for the duration of the experiment.

### Preparation of Petri dishes

Petri dishes are filled with defined media (DM) in which peptone found in the standard nematode growth medium [34] is replaced with defined amino acids and trace metals to minimise a batch-to-batch variation in peptone as previously described [25] with vitamin B_12_ added [35, 36]. Petri dishes for imaging are poured with DM agar 3 days before the worms are added. FUdR is added at a final plate concentration of 2 µM to prevent progeny production in all strains that are not genetically temperature-sensitive sterile. All other compounds are made up in a 100 × stock in required solvent and added to the agar before plates are poured. On the next day, Petri dishes are seeded with 100 µL of an overnight culture of OP50 *E. coli* in LB. Petri dishes are stored at 20°C with controlled humidity until 24 h after bacterial seeding, when they were transferred to an incubator at 24°C.

### Imaging on the WormGazer™

For each camera, images are taken every 0.8 s for a period of 160 s to create a group of 200 images and stored on a single-board computer. These images are converted into a series of analytical images on the single-board computer, and those images are transferred to a central server. After 5 min, the process is repeated. From these analytical images, the number of moving objects is calculated by applying a threshold of the minimum speed of each object of 10 µm s^−1^. The speed is derived from the length of the object divided by the 160-s time interval of the imaging window. Plates were censored if they failed a quality control inspection after the experimental runtime, for example if they were contaminated with another microbe or the worms had burrowed into the agar. Censored plates were omitted from data analysis.

### Manual lifespan protocol

SS104 *glp-4(bn2)* worms are prepared in the same way as for healthspan experiments. L4 worms are selected and picked onto Petri dishes with 30 worms per dish and 12 dishes per condition. Worms are scored every second day during weekdays from day 7 onwards and marked as alive, dead or censored. Worms are prodded with a platinum pick to check for movement before being marked as dead. Worms are transferred on days 7 and 14. Censored indicated the worms had bagged, burst vulvas or crawled off the plate. Kaplan–Meier curves are generated on GraphPad Prism and statistics on JMP statistical software.

### Statistical analysis

The shading on the time-series curves is 1 standard error of the mean across the curves for each Petri dish within the condition. The error bars on the bar charts are ± 1 standard error of the mean across the measurements for each dish within the condition. When comparing two conditions on a bar chart, the difference between the means is calculated, and the standard error on this is calculated with reference to the standard error on each individual measure, using Gaussian error statistics. The significance thresholds are then set with reference to the difference expressed in terms of its standard error. A difference of less than 1.64 standard errors is marked as not significant (ns). A difference between 1.64 and 2.33 standard errors is marked as one star (*), corresponding to *p* < 0.05 on a one-sided test. A difference between 2.33 and 2.83 standard errors is marked as two stars (**), corresponding to *p* < 0.01 on a one-sided test. A difference greater than 2.83 standard errors is marked as three stars (***) corresponding to *p* < 0.002 on a one-sided test.

### Supplementary Information

Below is the link to the electronic supplementary material.

Supplementary file1 (PDF 443 KB)

Supplementary file2 (MP4 2589 KB)

## Data Availability

Data used to plot the graphs in this article are available upon request.
