# Supplementary material for: Rapid measurement of ageing by automated monitoring of movement of C. elegans populations
Source: GeroScience. 2023 Nov 8;46(2):2281–93. doi: 10.1007/s11357-023-00998-w (PMC10828257; doi:10.1007/s11357-023-00998-w)
Supplement: Supplementary file 1 — Supplementary file1 (PDF 443 KB) [file 11357_2023_998_MOESM1_ESM.pdf]

Supplementary Data

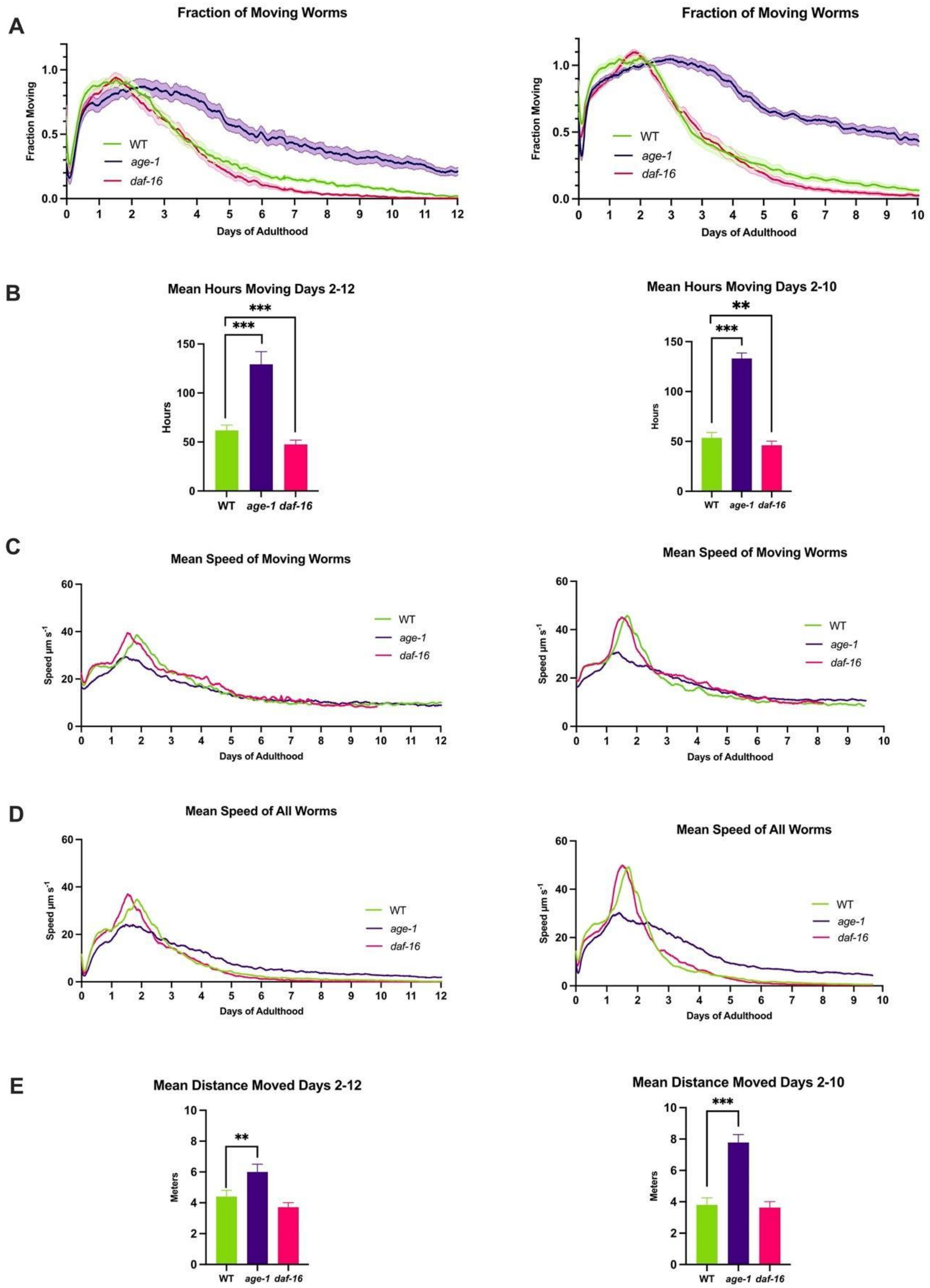

*Supplementary Figure S1 Direct Comparison of Two Repeats of age-1, daf-16 and WT worms. (A) The fraction moving graph shows the proportion of worms moving during the imaging window. (B) The area under the curve integration from day 2 to end of experiment for (A). (C) The mean speed of moving worms. (D) The mean speed of all worms, which is a function of A and E. (E) The area under the curve integration from day 2 to end of experiment for (D). Lefthand column; n ≥ 298 worms, 10-12 petri dishes per condition. Righthand column; n ≥ 420 worms, 14 petri dishes per condition. Plates with 2 µM FUdR on DM. *** = p <0.002, one-tailed test. Plotted in GraphPad.*

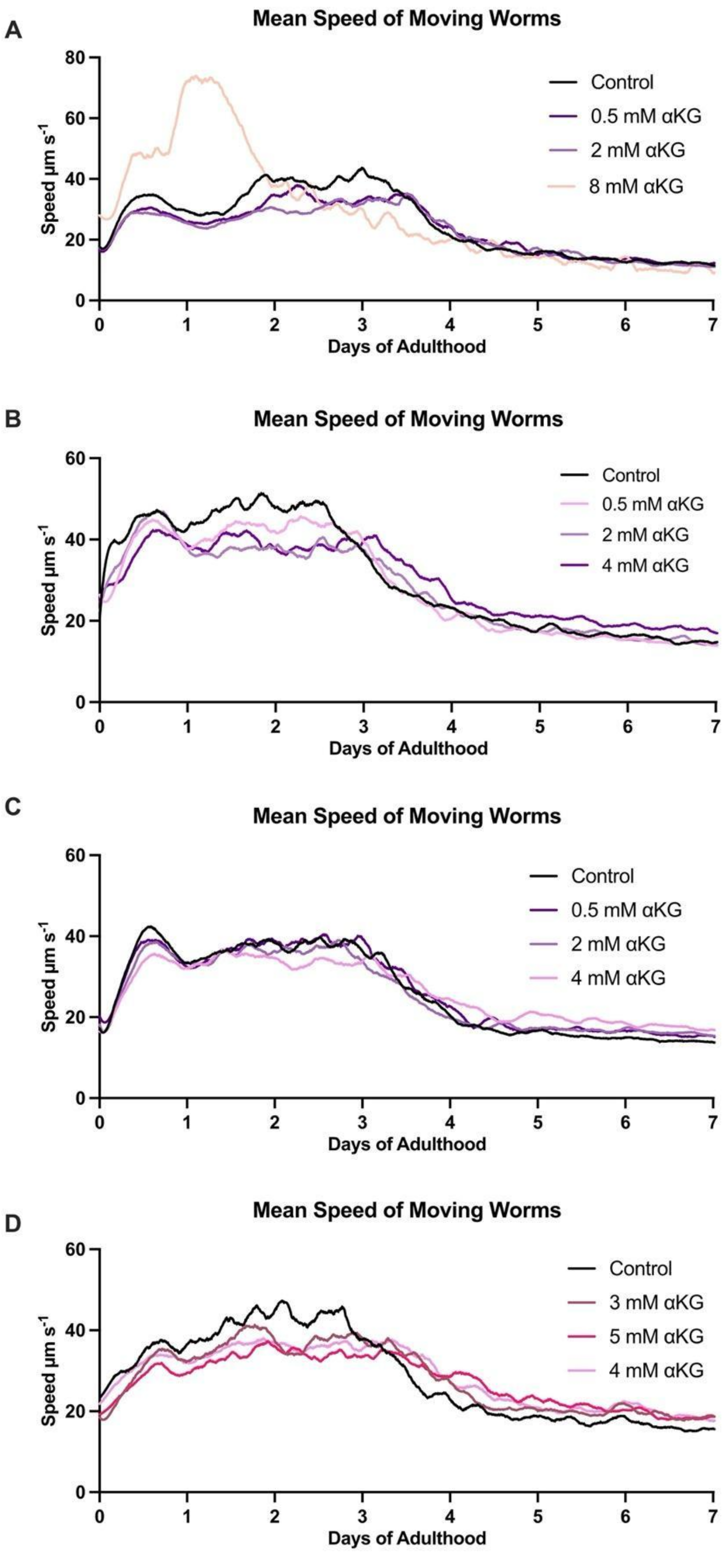

Supplementary Figure S2 Mean Speed Moving Worms for Alpha-ketoglutarate. Graphs are in order of appearance in Fig. 4. (A) Mean speed of moving worms. $n \geq 180$ worms, 6-8 petri dishes per condition. (B) Mean speed of moving worms. $n \geq 330$ worms, 11-12 petri dishes per condition. (C) Mean speed of all worms. $n \geq 300$ worms, 10-11 petri dishes per condition. (D) Mean speed of moving worms. $n \geq 150$ worms, 5-9 petri dishes per condition. Compound added to agar. Glp-4 worms used. Plotted in GraphPad.

Supplementary Table S1 Raw Data for the Percentage Comparisons for age-1, daf-16 and WT. Data taken from machine export files.

**Condition**	**Fraction Moving AUC Days 0.5-2**	**Mean Speed Moving Day 2**
Control	31.62 ±1.30	0.89 ± 0.05
age-1(hx546)	27.78 ± 1.87	0.85 ± 0.06
daf-16(mu86)	30.48 ± 1.34	0.86 ± 0.04

Supplementary Table S2 Manual lifespans were carried out at 24˚C. Worms were checked every other day on weekdays after day 7. SS104 glp-4(bn2) worms used, with OP50 E. coli on DM plates. Work carried out with assistance from Adelaide Raimundo. Statistics by JMP. All comparisons against control.

**Condition**	**n**	**Censor**	**Mean Lifespan**	**Standard Error**	**% Change**	**p-value (Log-Rank)**	**p-value (Wilcoxon)**
Control	290	100	18.39	0.28	-	-	-
1 µg/mL SMX	276	107	20.28	0.28	+10.27%	< 0.0001	< 0.0001
4 µg/mL SMX	276	108	20.99	0.36	+14.13%	< 0.0001	< 0.0001
8 µg/mL SMX	262	124	21.14	0.36	+15.00%	< 0.0001	< 0.0001

Supplementary Table S3 S2 Raw Data for the Percentage Comparisons of Mean Speed of Moving Worms in Automated Lifespan. Data taken from machine export files.

**Condition**	**Mean Speed Moving Day 2.5**
Control	49.68 ± 2.12
1 µg/mL SMX	40.39 ± 1.46
4 µg/mL SMX	34.37 ± 1.39
8 µg/mL SMX	32.48 ± 1.37

Supplementary Video 1 Movie showing worm movement, fraction moving and integration of the area under the curve between day 2 and day 14 for *age-1(hx546)* and WT. Movies from two representative petri dishes were compiled from the high contrast images from the WormGazer technology where the white tracks depict movement in each 160 second imaging window and the red tracks show historical movement. The fraction moving graph (top left) shows a smoothed curve of the fraction of worms moving within the imaging window. The integration of the area under the curve (bottom left) represents mean hours moving between day 2 to day 14, showing a statistically significant difference in movement between the strains. Full experiment in Figure 2 and Supplementary Figure S1
